# The Limits of Empowerment: How to Reframe the Role of mHealth Tools in the Healthcare Ecosystem

**DOI:** 10.1007/s11948-019-00115-1

**Published:** 2019-06-06

**Authors:** Jessica Morley, Luciano Floridi

**Affiliations:** 1 Oxford Internet Institute, University of Oxford, 1 St Giles, Oxford, OX1 3JS UK; 2 The Alan Turing Institute, 96 Euston Road, London, NW1 2DB UK

**Keywords:** Empowerment, Digital health technologies, Digital companions, Medical paternalism, mHealth, NHS

## Abstract

This article highlights the limitations of the tendency to frame health- and wellbeing-related digital tools (mHealth technologies) as *empowering devices*, especially as they play an increasingly important role in the National Health Service (NHS) in the UK. It argues that mHealth technologies should instead be framed as *digital companions*. This shift from empowerment to companionship is advocated by showing the conceptual, ethical, and methodological issues challenging the narrative of empowerment, and by arguing that such challenges, as well as the risk of medical paternalism, can be overcome by focusing on the potential for mHealth tools to mediate the relationship between recipients of clinical advice and givers of clinical advice, in ways that allow for contextual flexibility in the balance between patiency and agency. The article concludes by stressing that reframing the narrative cannot be the only means for avoiding harm caused to the NHS as a healthcare system by the introduction of mHealth tools. Future discussion will be needed on the overarching role of responsible design.

## Introduction

I want to ask what we will be talking about not when the NHS is 70, but when the NHS is 80. […] Well the first thing is we may well not be going to doctors for a diagnosis. […] we may well be in a world where if we show any symptoms of a disease we consider that a sign of failure. […] And accompanying all of that is likely to be a big shift in power from doctor to patient as patients use technology to put themselves in the driving seat of their own healthcare destiny, in the same way that we use technology to give ourselves much greater control over every aspect of our lives.

The previous quote is taken from then UK Health Secretary Jeremy Hunt’s keynote speech at the National Health Service (NHS) Expo Conference (the national conference for health innovation) in September 2017. It provides a succinct summary of the argument currently underpinning the *Empower the Person* digital strategy of NHS England,^1^ namely that mHealth tools (apps, mobile phones, patient monitoring devices, personal digital assistants, software as a device or other wireless devices^2^) will ‘empower’ individuals with the data they need to be proactive about preventing ill health.

**Fig. 1:**
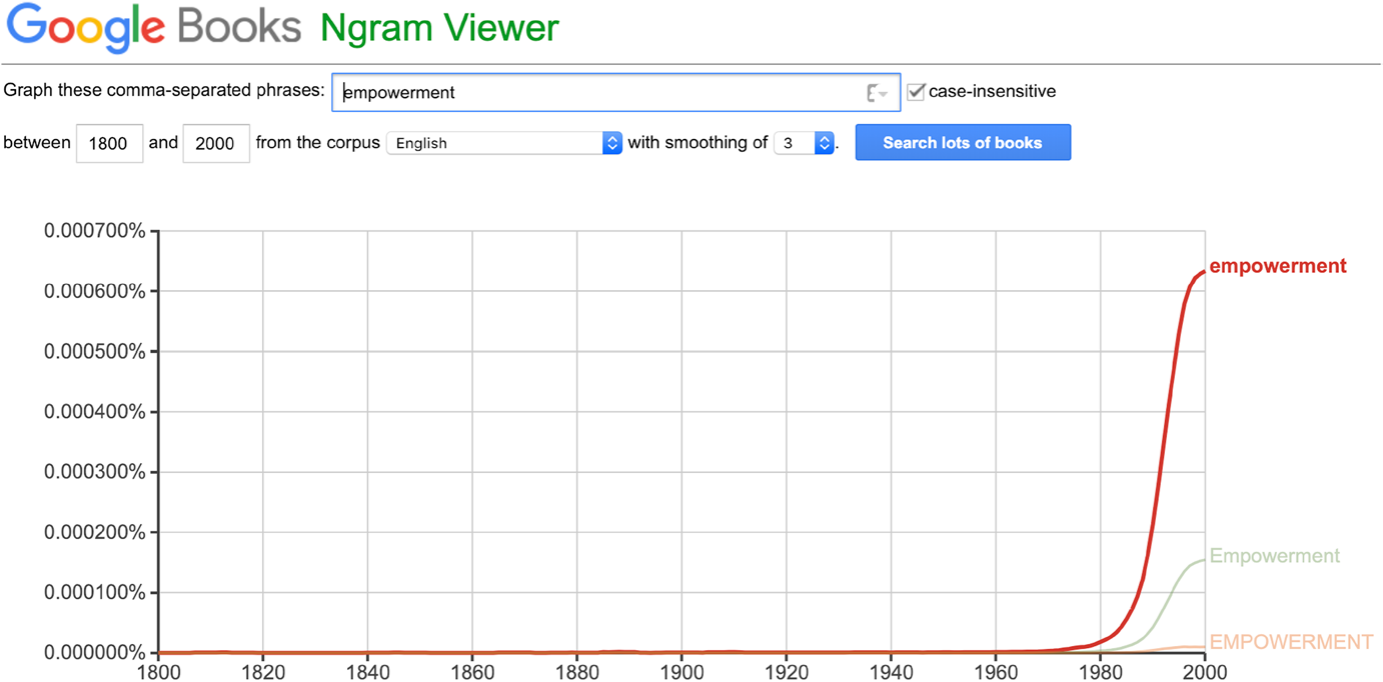
Ngram of “empowerment”, not case-sensitive, on Google English Corpus 1800–2000

The rhetoric of ‘empowerment’ is relatively recent, appearing first in the late 1970s and growing in popularity over-time, as an increasingly diverse range of fields became infatuated with the concept, from popular psychology to international development (Calvès 2009). Figure 1 highlights the rapid rise of empowerment as a cultural phenomenon, showing it peaking in the late 90s- early 00s when—as Anne-Emmanuèle Calvès (citing Wise 2005) points out—there was even a book published on self-empowerment for dogs. Today, it is still particularly strong in the health care sector, but can also be found operating in any context where citizens, customers, users, employers, or more generally end receivers of a good, a service, or some other kind of interaction are supposed to be placed in control, enjoy more autonomy and self-determination about their choices, and be able to represent and act upon their legitimate interests.

In the context of healthcare, the popularity of the empowerment narrative has led to a proliferation of research on the ways in which mHealth (Rich and Miah 2014) can be used to put individuals in control of their health (Lupton 2014), with researchers, technology providers and policy makers claiming that empowered patients will be more independent, have a better quality of life, and even adapt better to existing health conditions (Bravo et al. 2015). As a result, mHealth tools have come to be presented in a techno-utopian manner, which may go as far as portraying self-tracking as the panacea for preventive medicine (Smith and Vonthethoff 2017).

The issue with the all-pervasive nature of this narrative is that mHealth tools sit at the junction between philosophy and technology (Wagner 2018), resulting in what Lucivero and Prainsack (2015) refer to as the lifestylisation of healthcare. Apps, wearables and medical device software represent an exciting array of new tools for healthcare management (Federica Lucivero and Jongsma 2018), but they are also diverse technologies that, as active sociocultural products, are unlikely all to have the same ‘empowering’ effect on all individuals. Arguing that this is the case can be seen as deceptive, as these technologies are also constitutive components of the informational turn in sociopolitical power (Floridi 2015) and essential for the creation of an environment in which individuals can even be perceived as being complicit in their own self surveillance (Rich and Miah 2014).

Although there is a clear need to unpack the meanings and the uses of mHealth tools, there has been a lack of detailed social research (Lupton 2013) in the policy arena. Indeed, it would seem that the empowerment narrative has quietly permeated all levels of healthcare policy,^3^ without much attention having been paid to either the unevidenced assumptions on which it rests (Vezyridis and Timmons 2015), or the ethical concerns related to mHealth (including the empowerment claim) raised by several scholars (Lucivero and Jongsma 2018; Mittelstadt et al. 2014). This has led to oversimplified exaggerations about the benefits of digital health that fail to account for the variations in individual health needs, concerns, circumstances, behaviours, and digital literacy levels (Vezyridis and Timmons 2015; Smith and Vonthethoff 2017), and for how these varying individuals are affected by being subjected to normative constructs of patienthood, prevention, and illness. There is a risk that—without asking difficult questions about the power dynamics involved (Starkey 2003) and the ways in which the empowerment narrative might have negative ramifications (Spencer 2015)—the concept will become fetishized to an extent that impedes the real potential of mHealth tools (Archibald and Wilson 2011).

Clearly, there is a pressing need to identify and evaluate the assumptions underlying the empowerment narrative, as it exists in healthcare policy, in a way that highlights its ethical challenges (Lupton 2012). This is the task of the following pages. More specifically, the next section outlines the roots of the word ‘empowerment’ and its political history as a concept in healthcare, in order to ground the analysis of some ethical and methodological issues. The ‘Empowerment and the Digital Medical Gaze’ section introduces the concept of the Digital Medical Gaze to explain how mHealth tools can be seen as technologies of the self, making it possible to highlight the problems that this creates. ‘The empowerment marketing campaign’ section elucidates the value-laden nature of the empowerment narrative. This is followed by the ‘Doomed to Fail’ section which shows that the empowerment narrative rests on a fundamentally flawed assumption, namely that digital data, once acquired and transformed into information, will result in reasoned and reasonable decisions, ignoring the contextual complexities that moderate the ability of an individual or group (e.g., a couple, or a family) to interpret and act on the information they are reviewing. The following ‘Empowerment or Libertarian Paternalism?’ section further criticizes the empowerment narrative by questioning whether it truly represents a move away from paternalism. Finally, the ‘Digital Companions’ section presents an alternative conceptual model of mHealth tools that aims to overcome the issues inherent in the empowerment narrative, identified in the previous sections, and enables people and the healthcare system to capitalise on the opportunities presented by mHealth tools in a responsible manner. The conclusion highlights the risks the NHS faces if it chooses to stick dogmatically to the empowerment narrative and stresses how the benefits of the Digital Companions reframing will only be realized if the ecosystem in which they operate is responsibly designed with society-in-the-loop.

## The Roots of the Empowerment Narrative

The idea of empowering people to take better care of their health is not new. It has been present in neo-liberal health policy since at least the 1970s (Lupton 2013). However, in the UK, it significantly increased in popularity during the 1990s and early 2000s. It was (also) a reaction to the sustainability crisis faced by the NHS (Anderson and Gillam 2001; Lettieri et al. 2015). This prompted the Government to pivot towards a new proactive paradigm of healthcare provision (Moerenhout et al. 2018), focused on delivering ‘P4’ (personalised, preventative, predictive and participatory) medicine (van Roessel et al. 2017).

This paradigm shift coincided with the Internet becoming commercially available, leading to ‘info-liberal’ arguments (Catlaw and Sandberg 2018) that the amount of health information available on the Internet would lead to the emergence of a new generation of ‘expert patients’ (Henwood et al. 2003). The information would increase the self-esteem of these experts, empowering them to become active participants in their own healthcare (Wilson 2001), and resulting in them having a better quality of life (Fox et al. 2005). The arrival of mHealth technologies (including wearables, self-tracking software and health apps) and the subsequent explosion in the amount of data related to health, have enabled the logic of empowerment to gain more widespread and intense support than ever before (Lupton 2013).

Policy documents, such as *The Five Year Forward View* (2014),^4^*Personalised Health and Care 2020* (2014)^5^ (West et al. 2016), and most recently the *NHS Long Term Plan* (2019),^6^ have come to rely on the unquestioned belief that people who have unlimited access to their personal health data (van Roessel et al. 2017; Swan 2009) will take on the role of what Swan (2012) terms the ‘empowered biocitizen.’ These empowered individuals are framed as being able to acknowledge that they have both the responsibility (Wardrope 2015) for managing their own health, and the tools for doing so^7^ (Swan 2012). Goetz (2010) sums this up well in his description of the health decision tree:

Health is, in many respects, a system of inputs and outputs. The inputs start with the huge number of choices we make every day that have a great influence on our health: what we choose to eat, whether or not we exercise, how much we sleep, whether we heed our doctors’ orders. […] All of these inputs together create one primary output that is unique to us alone: our health, for good or for ill. […] The more we’re conscious of these inputs, the more often we take the time to think them through and maybe even write them down, the better are our chances of making the best decisions and having the best lives. (Goetz 2010, pp. xiii–xiv)

Yet, in all these policies, it is unclear *exactly how* access to data will empower people. This lack of clarity makes it almost impossible to evaluate (and therefore criticise and improve) how effectively the digital health interventions promoted by these myriad strategies ‘empower’ people to take control of their health. There is, therefore, a need to start with understanding what empowerment actually means and build from there to a more complex critical analysis of the way in which it is operationalised in digital health strategy. This is difficult because the term itself is poorly defined (Lettieri et al. 2015; Bravo et al. 2015; Tengland 2007, 2008) and—despite the fact that the vociferous use of ‘empowerment’ by politicians, policymakers, clinicians, and technology providers may seem to indicate otherwise—it is used both loosely and inconsistently (Roberts 1999) by all these groups. A scoping review completed by Bravo et al. (2015) found that even the most commonly used definition was still only used in 11% of the literature they reviewed.

This confusion can be blamed on the fact that the concept has no single point of origin. Starkey (2003) highlights that it stemmed simultaneously from the civil rights, self-help, consumerist, anti-racist and women’s movements. As a result, it is embedded in a range of competing discourses, including the consumerist and liberational models, which, respectively, highlight the need to give people choice and the opportunity to change their position in society (Starkey 2003).

To avoid this confusion also pervading this discussion, the next section clarifies the way in which NHS England’s *Empower the Person* digital strategy uses empowerment and introduces the concept of the ‘Digital Medical Gaze.’

## Empowerment and the Digital Medical Gaze

Almost all of the primary conceptualisations of empowerment outlined in the previous section are in use in the wider health promotion discourse (Sheehan 2014). However, as leveraged by info-liberal NHS policy, the *Empower the Person* narrative positions empowerment as an inherently performance-based, outcome-focused transformative process (Bravo et al. 2015; Tengland 2007, 2008). Garcia et al. (2014) describe this process as a micro-cycle of self-reflection consisting of four steps:

1. gaining knowledge [e.g. individuals read GP records and gain knowledge of their current state of health];
2. gaining awareness [e.g. individuals monitor steps and become aware of how active they are];
3. self-reflection [e.g. individuals reflect on how many calories they consume daily in relation to their daily activity level and what this might mean for their current state of health as outlined in their GP records];
4. action [e.g. individuals decide to walk to work instead of driving].

This process turns the individual in question into a reflexive patient (Henwood et al. 2003), making them responsible for creating their own self (Smith and Vonthethoff 2017). When this process occurs within the digitised healthcare sphere, this self is created through what may be called the *digital medical gaze*. First, the individual separates into a series of data flows, which are aggregated into a transparent (Floridi 2014) data double (Haggerty et al. 2000) ‘self’ that the individual can reflect on through a digital health tool (Floridi 2014). The individual uses this process of reflection to determine whether their digital self would be perceived by a medic as healthy or unhealthy. Second, this close interrogation (Catlaw and Sandberg 2018) acts as a trigger for intervention (Ruckenstein 2014) by the individual on her or his self to shift the self in the desired ‘healthy’ direction.

From this perspective, allegedly empowering mHealth tools can be seen as technologies of the self (Floridi 2014). Subsequently, the act of reflecting on one’s self through the digital medical gaze can be seen as a Foucauldian practice of care of self, where one’s faults and limitations are observed, and attempts are made to transcend them (Catlaw and Sandberg 2018). Michel Foucault notes that this process of surveying faults is one that is inherently linked to power. As such, the data generated by mHealth tools are imbued with constructed and constructive power and knowledge^8^ that bring the digital self into being, so that it can be acted upon (Nagington et al. 2016).

Proponents of the view that intentionality is the key factor in ethical evaluation (including those who believe ‘the end justifies the means’) (Floridi 2016a) may believe that, if this process of reflecting on one’s self through the digital medical gaze results in an individual experiencing a healthier quality of life, then it is not ethically problematic. To demonstrate why the practice is still concerning, even if the intention of promoting self-surveillance is to improve people’s health outcomes, it is necessary to question which health outcomes are being promoted, why, and what the implications of this are. This is the purpose of the following section.

## The Empowerment Marketing Campaign

In order to highlight to the individual what their faults and limitations are, with the intention of encouraging attempts to transcend them (Catlaw and Sandberg 2018) most mHealth tools, leveraged in the ‘Empower the Person’ narrative, work by establishing a baseline for ‘health’ in one domain (e.g. number of calories to be consumed in a day, or number of steps to be made) and highlighting to the individual how far their self is away from that baseline, through the digital medical gaze. Policymakers and politicians supporting this approach would have people believe that these baselines are always based on ‘objective knowledge’ (McLaughlin 2016). However, the reality can be more opaque or unclear, and sometimes imposing some kind of scientific credibility glosses over the fact that it is those in either authoritative (clinical) or political positions of power that set the baselines (Spencer 2015). These individuals may themselves be victim of mistaken assumptions, but they may also have distinct goals in mind when they set these baselines (Roberts 1999), which means there may be an avoidable, yet embedded and invisible agenda shaping (perhaps even inadvertently) the information that is being used to ‘empower’ in any given situation. The notorious case of the ‘10,000 steps a day recommendation’ is indicative. It has become normative despite the fact that it is based on the very simple logic of ‘some physical activity a day is better than none’, which actually means that the number (and intensity) of steps that need to be taken above and beyond an individual’s standard level of activity to be beneficial is highly variable (Tudor-Locke et al. 2011). Chance, poor science, mere fashion, group behaviour (‘everybody does it’) and the pressure to quantify precisely and universally about vague or fuzzy concepts that should be tailored individually may end up shaping the narrative of health.

When this gloss is removed, the value-laden nature of the baselines used to determine whether the self is ‘healthy’ becomes obvious (and thus so does the value-laden nature of the *Empower the Person* strategy). As McLaughlin (2016) points out, it is impossible to promote one type of ‘healthy’ behaviour over another without having a firm value-driven opinion on what the good life looks like and how people should behave in order to achieve it. The risk of embedding such ‘healthist’ values in supposedly empowering mHealth tools lies in disciplining (or, alternatively, frustrating and marginalising) those with perceivably inferior moral beliefs about health (Lupton 2013) until they meet the standards of the effectively-marketed healthy ideal type (McLaughlin 2016; Grace 1991; Williams 1984). Note that the problem is further exacerbated by the visualisations of unrealistic bodily appearances promoted by the advertising and fashion industries.

Within the digital health sphere, or the NHS’s digital ecosystem, these values may be internalized (Galič et al. 2017) and reinforced by push notifications, buzzes, and other gamification features of mHealth tools that are supposed to be motivating. However, they may also ensure that individuals become complicit in their own self-surveillance (Rich and Miah 2014). This is ethically concerning because of the implications for moral responsibility.

Moral responsibility involves both looking forward, where an individual is perceived as being in charge to ensure that a desired outcome is achieved (as described above) and looking backwards to appropriate blame and possibly redress, when a failure has occurred (Wardrope 2015). Healthcare has always involved both these elements of responsibility. For example, if given an antibiotic prescription for tonsillitis, an individual has forward-looking moral responsibility for completing the course to ensure the infection clears, and backward-looking responsibility if the infection recurs because they did not take all the doses. Empowering mHealth tools are seen as affording individuals’ greater visibility of the processes of the self, which combines with existing free will (Flaskerud 2014) to produce an increase in responsibility (Vezyridis and Timmons 2015; Baistow 1994).

This increase in responsibility is symbolised in the replacement of ‘patient’ with ‘user’ in the ‘User-Led NHS’ healthcare policy narrative (Fox et al. 2005), which accompanies that of ‘Empower the Person.’ At face value, this switch may seem irrelevant. However, as McLaughlin (2016) states, different labels conjure up different identities, and whilst a patient identifies with Parson’s sick role, i.e. a passive individual devoid of responsibility (Roberts 1999), a user^9^ is pressurized to identify as an active and engaged individual (Neuberger 1999), capable of being held responsible for their health.

Through this process, which Daniel Goldberg (2012) terms ‘methodological individualism’, the digital self, created through the digital medical gaze, risks being enslaved rather than empowered (Murray 2007). This risk arises because the empowerment narrative has, what Eric Juengst and colleagues (2012) term a ‘correlative vice’, whereby it can feel like an elaborate mechanism for victim-blaming (McLaughlin 2016; Danis and Solomon 2013) that denies^10^ the fact that much of health is controlled by macro forces over which the ‘user’ has only very marginal or no control (Riger 1993; Green and Vogt 2016). This is in contrast to the argument, made by Per-Anders Tengland (2012), that empowerment is (in general) less likely to result in victim blaming than behaviour change models and is a growing concern, as the range of data that the digital gaze is expected to reflect upon expands to include genotypic as well as phenotypic information (Green and Vogt 2016).^11^

Direct to consumer genetic tests have been available for purchase since 2007 (McGowan et al. 2010) and, despite currently being used as another means of providing authoritative justification of actions by clinicians (Juengst et al. 2016), are also often marketed under the banner of patient empowerment (Juengst et al. 2012). Proponents argue that—when these data are included in electronic medical records (such as that available through the NHS App^12^) (Hazin et al. 2013), and combined with the phenomic data (Juengst and McGowan 2018) generated by wearables—a tipping point will be reached when P4 medicine becomes a reality (Vegter 2018). The logic underpinning this argument—that individuals will be able to act upon the genomic and phenomic contributors to disease (Foster and Sharp 2008)—denies the fact that the quality of these data is questionable (Green and Vogt 2016) and interpreting the information held within requires an unusually high level of health literacy (McAllister 2016). Unless tackled before open personal health records that combine all these data become a reality, this denial and over confidence (James 2014) in the imagined affordances (Nagy and Neff 2015) of P4 medicine, will trap people in a quasi-contract where they will be expected to achieve unrealistic expectations of ‘wellness’ (Juengst and McGowan 2018), and labelled as irresponsible citizens (bad users) when they fail (Juengst et al. 2012). This creates a scenario in which responsible users who are unsuccessfully empowered are considered bad users (Scott 2018) or cyberchondriacs (Lewis 2006; McMullan et al. 2019).

This (not-too-distant) future scenario, in which people may have large volumes of complex information thrust upon them in the name of empowerment (Johnsson and Eriksson 2016) might seem extreme when such action is often ethically justified on the basis of respect for persons (Danis and Solomon 2013) and their autonomy (Chiapperino and Tengland 2015). However, denying the possibility of this future creates a situation where it is possible for healthcare policymakers to turn a blind eye to the potential for mHealth tools to be ineffective or drivers of increases in inequality of health outcomes (Wardrope 2015). Thus, to help override this denial, the following section highlights the difference between *procedural* and *relational* autonomy, showing that, whilst it is the former that the *Empower the Person* strategy promotes, it is actually the latter that dictates a person’s genuine ability to improve their health.

## Doomed to Fail

The ‘Empower the Person’ strategy and associated policies seem to be predicated on the assumption that, as long as people are able to look at their data self through an mHealth tool, they will be able to make a rational and reasoned decision about what to do next. This is based on a rather narrow definition of autonomy, known as *procedural autonomy*, and it puts all the attention on the decision rather than the action that follows the decision (Owens and Cribb 2017). In reality, for mHealth tools to be ‘empowering’ they have to result in some form of behaviour modification intended to improve the self (Catlaw and Sandberg 2018). This puts the emphasis on the broader *relational autonomy*, which centres on action rather than decision^13^ (Owens and Cribb 2017). Accounts of relational autonomy recognise that acting is more complicated than deciding (Owens and Cribb 2017), not least because it takes place within the ‘health habitus’, where the types of online health information that people interact with, the ways that they interpret it, and the options that they have to act on it (Lewis 2006) (Mittelstadt et al. 2014) are all constrained by a variety of socio-economic factors that affect some groups more than others (de Freitas and Martin 2015).

Constraints on the actions triggered by people’s digital medical gaze can be related to their gender, race (Lewis 2006), income and education levels (Yeo 2016), confidence in digital and health literacy skills (Schaffler et al. 2018), or, most likely, a combination of all of these factors.^14^ This is because mHealth tools do not operate in a vacuum. They are used by people embedded in larger social structures (Visser et al. 2016) that can considerably hinder or enhance their ability (McAuliff et al. 2014) to meet the established standards. This supports Owens and Cribb’s (2017) assertion that there is reason to be highly sceptical about the claims that mHealth tools offer people genuine opportunities to improve their health. Instead, they could exacerbate existing health inequalities, by creating a scenario in which backward-looking responsibility (blame) is placed on ‘bad users’ for whom it would have been almost impossible to achieve the defined standards of health in the first place.

All of these reasons combined make it clear that empowerment-based healthcare policies, such as ‘Empower the Person’, have serious conceptual, ethical, and methodological problems (Williams 1984). This does not mean, however, that policymakers who believe in the benefits of mHealth tools are insincere. mHealth tools can be used to provide significant efficiency gains to the NHS (Rich and Miah 2014) which, in turn, can release resource for those who are less technologically-enabled or who require more significant medical intervention (Watson 2018). In addition, digitally provided information can enable those with the right capacities (Wagner 2018) to ask more relevant and accurate questions, so that they feel more involved in planning for their health (van Roessel et al. 2017). This is the case particularly for those living with long-term conditions, who increasingly rely on digital health interventions, such as those within the ‘Internet of Healthy Things’ network, to maintain independence (Wakefield et al. 2018). Furthermore, mHealth tools can be designed better, and in ways that can avoid or minimize some of the issues analysed above, for example by providing more flexible ways to tailor recommendations to users’ specific needs.

Thus, just as it is incorrect to present a digitally-enhanced NHS as a techno-utopia, it is equally incorrect to present it as a techno-dystopia. It is the ‘empowering’ rhetoric that is outmoded, inaccurate, and in need of replacement, not the digital tools themselves and their potential or actual uses and benefits. However, before we look at what might be a suitable alternative, it is important to ensure that the lessons from the past are learnt (Giordano 2010). This is the task of the next section.

## Empowerment or Libertarian Paternalism?

When the NHS was first outlined in the 1942 *Beveridge Report*, disease was presented as a social evil and its control was seen as being essential to the new post-war social order (Anderson and Gillam 2001). At the same time, the typical doctor-patient relationship of the age was inherently paternalistic, with doctors being presented as unequivocally right (Matthews 1986) about patient needs. This rendered patient input largely irrelevant (Waithe 1983; Charles et al. 1999). Thus, the NHS of 1948 produced the ‘nanny state’ (Flaskerud 2014), by combining the newly paternalist state with the already inherently paternalist medical profession (Anderson and Gillam 2001). This remained the status quo until, over the course of 30 years between the 1960s and 1990s (Aggarwal et al. 2014), both Margaret Thatcher’s Conservatives and Tony Blair’s New Labour shifted healthcare policy towards the ‘informed model’ (Charles et al. 1999), which centred on the transfer of information between the two parties (McLaughlin 2016). This remains the model on which ‘Empower the Person’ is built, which potentially leaves critiques of the narrative, such as the one presented in this article, open to accusations of advocating for a return to paternalism. To understand whether this is a valid criticism, it is necessary to consider whether mHealth tools, in their current framing, have truly moved the healthcare model away from its paternalist origins.

To do this requires revisiting Parson’s sick role concept which, as stated earlier, presents ill individuals as submitting passively to the administrations of their doctor (Roberts 1999). This passivity is justified on the basis that all illness represents a state of diminished autonomy. This diminishing of autonomy renders the ill person dependent on their doctor, who acts paternalistically to return them to their previous autonomous state (Komrad 1983). Thus, autonomy and paternalism are not seen as being diametrically opposed and incompatible, but rather relational and dependent on context. This point will be important later on, when looking at how to reframe the digital health narrative. The digital health realm is a specific context in which the institutionalized ‘healthy’ behavioural norm obligates people not only to strive to maintain their health but also to see themselves as incomplete (or ‘sick’) and in need of continuous improvement (Catlaw and Sandberg 2018; Juengst and McGowan 2018). Consequently, mHealth tools can be seen as ‘hyper-nudging’ (Yeung 2016), whereby reduced autonomy of the digital self, constantly striving for self-improvement, is supplemented by libertarian paternalistic algorithms that alter the presentation of the digital self to ‘nudge’ (Thaler and Sunstein 2008) the individual into taking pre-determined actions (Snowdon 2018). From this perspective, even subtle changes to the way in which patient education information is presented can be seen as being unduly persuasive (Reach 2016).

This link between paternalism and nudging is not uncommon. However, in the offline world, Thaler and Sunstein have argued that, as long as the nudging is done in a transparent way, so that the person being nudged is aware that it is happening and why, then it cannot be seen to be done against the person’s consent and therefore cannot be paternalistic (Snowdon 2018). This is already questionable in an offline context: transparent paternalism is no less paternalistic than the opaque one. The fact remains that the more successful structural nudging approaches (the type of nudging being referred to here where the actual nature of choices is altered to produce de facto compliance) are in shaping an individual’s behaviour, the less respectful they are of that individual’s choices, and thus the more paternalistic the practice is (Floridi 2016b). Now, there may be good reasons to be paternalistic: if someone wants to jump in front of a train, respect for their choice would seem odd. Likewise, nudging might have to be paternalist in cases in which ill and irreversible consequences are in place. But all this should not mask the fact that nudging is intrinsically a paternalistic strategy to shape someone’s behaviour or choices. And such a relationship is further problematic in a world that increasingly erases any boundary between online and offline, and make people live ‘onlife’ (Floridi 2014), and in a black-boxed world (Pasquale 2015; Schmietow and Marckmann 2019) of digital health, which is becoming increasingly automated, and where transparency may not be made available. Nudging when people are living ‘onlife’ (Floridi 2014) can represent a dangerous form of illiberal manipulation (Floridi 2016b) whereby the digital self, the source of self-reflection in the digital medical gaze, is constantly being algorithmically reformulated^15^ to enable different nudges, promoting obedience (Spiekermann and Pallas 2006) and undermining the integrity of self (Cheney-Lippold 2017), in ways that are even less perceivable than in the analog world.

The issue is that, as DuFault and Schouten (2018) stress, for individuals to be able to exert agency over these data-driven suggestions they would need to have some good understanding about the underlying data, processes, and technical possibilities. This is an understanding that the vast majority of the population simply does not have. For example, The National Literacy Trust estimates that in 2018 16.4% of adults in England, or 7.1 million people, were functionally illiterate, let alone digitally savvy. It is, therefore, reasonable to question whether an individual is genuinely able to exert agency in this context, especially as within the NHS they are not able to choose to ‘take their business elsewhere’ (Snowdon 2018), challenging the notion of an autonomous and free subject (Murray 2007).

Thinking through these implications and how to reframe the positioning and design of mHealth tools is getting increasingly important^16^ as we hurtle towards the world of an algorithmically-led (or artificially intelligent) NHS, in which ubiquitous computing (Spiekermann and Pallas 2006) and the Internet of Healthy Things are the norm. In this environment, the threats to integrity of self (where individuals are unaware of the forces acting on their self (Cheney-Lippold 2017) become magnified and, given that damage to a person’s psychological integrity, can be perceived as a ‘harm’, not accounting for this potentiality, poses the risk of creating a system that violates the first principle of medical ethics: *primum non nocere* (first, do no harm) (Andorno 2004). However, if the risks are accounted for (Gilmartin et al. 2018), one *could* shape the digital health ecosystem so that it enhances human capabilities (Loi 2018) rather than constrains them unduly. The question, to be addressed in the next section, is, if the paternalistic model was rejected and the digital information model with its empowering rhetoric has failed to be non-paternalistic (Reach 2016), what other models are there that will enable us to do this?

## Digital Companions

A third model for the doctor-patient relationship that has garnered a lot of attention since the 1990s is the shared-decision making model, or the partnership model, as illustrated by the NHS Executive publication of the *Patient Partnership Strategy* in 1996 (Anderson and Gillam 2001). Shared decision making seeks to deal explicitly with the fact that each partner brings a different kind of knowledge to the table. The clinician knows more about the quantitative and scientific aspects of the condition with which an individual may be living and their options for treating it, whilst the individual knows more about the qualitative and experiential aspects, the wider social, economic and environmental constraints within which they are living, and the impacts that a specific treatment plan may have on their life (Charles et al. 1999). The respective lack of knowledge in each party acts as a determining factor, limiting the options that they perceive to be plausible (Flaskerud 2014). Shared-decision making models insist on the equal exchange of this information so that it is possible for the two parties to reach a mutually agreeable decision. However, these models have been criticized for being heavy on theory and light on advice on how to translate the theory into practice (Anderson and Gillam 2001).

It would be almost impossible for shared-decision making models to be translated into practical action because the theory does not deal with the questions of agency and context. Almost always these ‘conversations’ are happening in a space that biases one type of knowledge over the other. A doctor’s office adds gravitas to the opinion of the clinician and the individual’s home where the conversation is between individual and digital health technology, or where the technology is mediating the conversation between clinician and individual, prioritizes the opinion of the individual. In neither scenario do shared-decision models provide guidance on who is ultimately responsible for taking action, once the knowledge exchange has happened. Either the clinician acts as agent (Charles et al. 1999), taking on board the knowledge provided by the individual and trying to make the decision that they think the individual would make for themselves, which makes the exchange paternalistic; or the individual takes on the knowledge provided by the clinician and is expected to act on it, bringing us back to the informed model underlying the digitally-empowered narrative. Since paternalism has been completely rejected, most instances of shared-decision making fall into the latter category and are thus no different from those that stem from the empowerment rhetoric.

Ultimately, all of these models have failed methodologically and ethically because they assume that the relationship between individual and doctor, mediated by a digital health technology or not, remains static. The reality is that multiple complicating factors related to individual preferences and organisational infrastructure combine to make this simply not the case (Lucivero 2017). Both Mark Siegler’s (1982) concept of the physician–patient accommodation and Thomas Szasz and Marc Hollender’s (1956) dynamic therapeutic model recognize this dynamism with both concepts, arguing for a more accepted recognition of the fact that the balance between paternalism and autonomy (or patiency and agency (Wagner 2018) should be constantly updated depending on context (Komrad 1983). This is an argument that has been validated numerous times. Studies by Dickens and Picchioni (2012), Rise and colleagues (2013), Covell et al. (2007), Schattner et al. (2006), Kraetschmer et al. (2004) and Levinson et al. (2005) all examine either how individuals wish to be classified (as patient, user or other suggested alternative) or the extent to which they want to be the ones in the driving seat of decision-making. All studies found that the answers to these questions are variable and contextually dependent, with multiple factors such as trust in clinician, newness or severity of symptoms, or confidence, all affecting the preferences in real time. This shows that the relationship between social factors, rationality and individual’s desire to be involved in making choices is complex (Walach and Loughlin 2018). Thus, there is never going to be a one-size-fits all model for the relationship between autonomy and paternalism (Carrard et al. 2016). Agency and patiency, in doctor-patient relationships, will always be dependent on the specific nature of the decision and the current circumstances of the individual in question (Whitney et al. 2008). Indeed, taking a meta-rights approach, if we give individuals the right to take a leading role in deciding how they want to be treated, it makes sense that we should also give them the right not to exercise this right (Basu 1984). As the Royal College of Surgeons states

if a patient makes a clear choice to follow a surgeon’s recommendation and not to be informed of the risks and benefits of alternative treatments then this is an exercise of autonomy.^17^

The real benefit of mHealth tools can be seen precisely in providing individuals and clinicians with the ability to navigate this difficult and ever-shifting terrain. By acting as external repositories for the wills and desires of the individual in different circumstances, as well as storing information about their wider circumstances, mHealth tools can act as volitional aids (Wagner 2018), which ensure it is the individual’s desire and potential for autonomy and agency that is respected (Dickens and Picchioni 2012), rather than assuming that they always wish to be empowered. This works in two ways.

First, it allows for a more flexible classification of the mHealth tools themselves, placing the role in which they put the individual along a spectrum that mirrors that developed by Szasz and Hollender (1956), from passive observer to active participant. This enables greater transparency about the true purpose of each individual tool and, importantly, does not prevent an individual from feeling empowered by using an mHealth tool that meets their current need, it simply means that they are not under pressure to feel this way.

Second, it puts the individual in control of the balance of power between their embodied qualitative knowledge and that of the clinician or artificially intelligent agent. For example, if they are an individual living with a long-term condition, such as diabetes, they may wish to be always the agent and may feel completely comfortable using wearables and software to monitor continuously their condition, without much or indeed any input from a clinician. However, if they are a person who lacks in confidence or digital literacy skills, or someone who is frightened by new symptoms, they may wish to use a digital diagnostic tool (a symptoms chatbot for example)^18^ that affords them the ability to express what is worrying them in an environment in which they do not feel judged, knowing that the results will be passed direct to a clinician, who can handle the conversation about what the results mean and what to do next. Equally, this can work the other way around. If a clinician, for example, has a recommendation about an individual’s diet, they can hypothetically send that information to the individual’s *My Fitness Pal,* which can access other information on an individual’s food preferences and budget constraints and make suggestions that meet both the individual’s and the clinician’s requirements, whilst being upfront about the fact that it has done so. As this does effectively involve an alteration of the choice architecture, this latter point of transparency is particularly important, if the mHealth tool is to avoid being accused of the illiberally manipulative form of nudging (Floridi 2016b) criticized earlier, but instead meet the requirements of wellbeing, enhancing ‘ethical manipulation’ (or informational nudging (Floridi 2016a)) often used by clinicians to gain informed consent when a patient asks for advice directly or lacks the capacity to choose rationally in a specific situation (Cohen 2013).^19^

When these criteria are met, the reframing repositions mHealth tools provided or promoted by the NHS as *digital companions* rather than empowering devices. This is not to imply that people become literal ‘companions’ with the technology (as suggested by Turkle’s 2011 interpretation of ‘Digital Companion), but that these technologies can allow for the outsourcing of difficult decisions (Smith and Vonthethoff 2017) about the constantly changing dynamic between individual and clinician (or clinical advice if the advice comes from a different non-human agent) and act to support both parties in the exchange of information. Experiments where this kind of interpretive flexibility has been enabled in the offline world have demonstrated the positive impact it can have. Valérie Carrard and colleagues (2016), for example, demonstrated that, when a GP was instructed to adapt different aspects of their nonverbal dominance depending on an individual’s expressed preference for level of paternalism during a consultation, the outcomes of the individual were considerably higher. This would indicate that reframing the digital health narrative from empowerment to companionship can ensure that the benefits of digital health tools are realized whilst minimizing some of the potential harms that have been discussed in relation to the empowerment and the paternalistic approaches.

It is possible to illustrate the potential success of this reframing by considering the example of the use of mHealth tools for those living with Cystic Fibrosis. This example also serves to highlight an important point that, whilst the analysis thus far has focused on the individual level i.e. the relationship between a single patient and their clinician (or clinical agent), it can also apply at the group level,^20^ for example by comparing the needs from mHealth tools of those living with a long-term condition and those without, and health care providers providing general advice (e.g. Pharmacists) and those providing specialist or emergency advice (e.g. Urgent Care specialists).

### The Use of mHealth Tools by Those Living with Cystic Fibrosis

Cystic Fibrosis (CF) is a genetic condition diagnosed at birth that causes a range of life-limiting symptoms that affect the whole body. It typically affects younger people (as the upper limit of life expectancy is 40) and can have a significantly detrimental effect on quality of life. However, if well-managed people with CF are successfully able to live relatively independent lives, without constantly having to rely on interventions from health care professionals (HCPs).^21^

The extent to which individuals with Cystic Fibrosis are able to manage their own care varies considerably over their life, depending on personal factors such as age, life-stage, emotional wellbeing and environmental factors such as where the individual lives and how much they earn (Floch et al. 2018). This means that for the individuals over the course of their life, the amount and type of information that should be taken into account when making decisions about their care (and who is responsible for making these decisions) varies considerably. In short, their potential for autonomy, and (Floridi 2016b) willingness to accept paternalistic intervention varies.

Due to these constantly changing needs, presenting mHealth tools targeted at these individuals, such as *CFBuzz,*^22^ as always-empowering devices is unrealistic and potentially harmful. Instead, presenting targeted mHealth tools as Digital Companions recognises that, just as people lean on their friends and family for different reasons at different stages in their life and need, the needs of those living with Cystic Fibrosis that can be met with mHealth tools will vary depending on the context. For example, when the individuals are young teenagers, just learning to manage their condition independently (high potential for autonomy), they might need more guidance, although be less willing to accept advice (low willingness to accept paternalism), and be less aware of how different environmental factors affect their condition and so may benefit from a Digital Companion that is able to give direct instructions, based on passive monitoring and data aggregation, presented in an explainable but non-paternalistic fashion. However, by the time the individuals are adult and accustomed to managing their own condition, they may need less input from their Digital Companion, and just require medication reminders or a calculator to help them calculate dosage if something about their daily routine has altered.

The European Union funded project ‘MyCyFAPP’ aims to recognise this by creating a suite of digital self-management apps, which provide access to information, motivate, and strengthen compliance, in ways that are flexible enough to deal with individual’s changes in life stage (e.g. age) and life changes (e.g. worsening of disease) and the associated adaptations in level of intervention (paternalism) required (Floch et al. 2018).

**Fig. 2:**
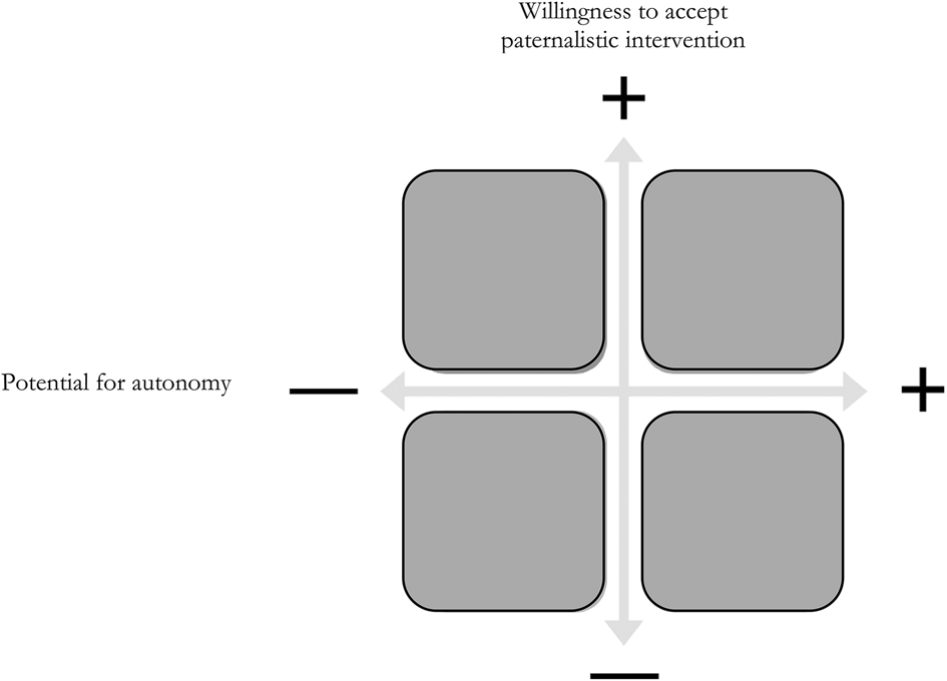
Illustrative conceptual model of mHealth tools as Digital Companions. To meet the Digital Companion criteria, mHealth tools must be able to move within the quadrants and across the quadrants depending on who is using them [Being consciously aware of this is necessary if the pitfalls of classic ethics where moral evaluations are assumed to be monotonic are to be avoided. In a dynamic multi-agent system (such as the digital health ecosystem) it is not possible to assume this level of stasis as each different type of interaction (any combination of person, group or artificial agent on either side of the equation) could produce a differently weighted moral outcome depending on the circumstance (Floridi 2016a)]

If the mHealth tools included in this collection were plotted on the conceptual model in Fig. 2, those aimed at children would be placed in the top left-hand corner whilst those aimed at adults would be placed in the bottom left-hand corner from an *intended* use perspective. However, the way that they are actually used by the individual, depending on the factors discussed, may mean that, occasionally, for example, an mHealth tool designed to meet the criteria of the bottom-right corner may be used in a way that meets the criteria of the top right-hand corner.

In none of these illustrative examples of mHealth tools for those living with Cystic Fibrosis is it assumed that the provision of an mHealth tool has automatically empowered the individual in question. Instead, in each instance the mHealth tool is used as a third variable (Floridi 2016a, b) to mediate the relationship between individual and HCP in a way that respects their willingness to accept paternalistic intervention, and maximises their potential for autonomy. This creates a symmetry of information, where each party has access to the same (relevant)^23^ information, and decisions can truly be shared (Durante 2014). In this sense, such examples of mHealth tools can be seen as integral parts of an environment that facilitates the maintenance of Millian liberty, in which the decision is made by the person who has the right to make it, even if that person (or artificial agent) is a third party (Coggon and Miola 2011). In other words, they form the infraethics of a responsible digital health ecosystem (Floridi 2014). This is important because, even though these isolated examples are seemingly innocuous, we have already seen how aggregated interactions between mHealth tools and individuals can result in a morally negative outcomes (i.e. many mHealth tools portrayed as being empowering has led to an ecosystem that assumes information = empowerment to a potentially harmful extent).

## Conclusion

Overall it can be seen that the empowerment narrative currently surrounding the use of digital health tools in the NHS arose from a desire to use them as a means of shifting the doctor-patient relationship away from paternalism and towards enhanced autonomy (Chin 2002), whilst reducing the costs of delivering in-person healthcare by trying to prevent ill health occurring in the first place and enabling those living with long-term conditions to do so independently. This is not a poor idea, but it goes too far. By calling the NHS digitisation project *“Empower the Person”* the existing digital health ecosystem is presented as a techno-utopia. Within this utopia, digital health tools are portrayed as being the enablers of the informed model of doctor-patient relationships, within which digitally provided information somehow releases individuals from all the other constraints that limit their ability to meet a narrow definition of perfect health, and makes them willing to accept full responsibility for their health as the price for being given complete (and static) autonomy. By sticking to this narrative, the NHS risks not being able to capitalise on the true opportunities presented by mHealth tools to act as digital companions to both patients and clinicians within the system, providing the individual with a chance to control their desire and potential for autonomy, and clinicians with the chance to present their recommended advice in a way that respects the individual and is interpreted within the context of their individual circumstances. The benefits of this reframing will, however, only be realised if the ecosystem for the development, deployment and use of mHealth tools is responsibly designed with society-in-the-loop (Rahwan 2018).^24^

## Footnotes

1 Policies across US and other European countries also focus on incentivising digitally-enabled ‘patient-centred’ solutions (Lucivero 2017), however, the focus of this article is on the strategy employed by NHS England as its centrality and national scale is unique and by calling the programme ‘Empower the Person’ NHS England is clearly signalling the significant reliance it has on ‘empowerment’ as a guiding narrative. The current (as of February 2019) Empower the person roadmap can be viewed here: https://indd.adobe.com/view/119c9ee5-6acb-4f52-80c2-d44fc03fdc91.

2 There is currently no agreed definition of what constitutes mHealth tools but these are the technologies that are most commonly referred to in relation to digital health which is why, with the exception of software as a device, they are the technologies explicitly referenced by the World Health Organisation in the results from its mHealth survey WHO Global Observatory for eHealth, & World Health Organization. (2011). *MHealth: new horizons for health through mobile technologies*. Retrieved from http://www.who.int/goe/publications/goe_mhealth_web.pdf.

3 Searching the social sciences citation index of the web of science for ‘empower’ AND ‘health’ returned 6651 articles in December 2018.

4 NHS England. (2014). Five Year Forward View. Retrieved from https://www.england.nhs.uk/wp-content/uploads/2014/10/5yfv-web.pdf.

5 HM Government, & NHS England. (2014). *Personalised Health and Care 2020 Using Data and Technology to Transform Outcomes for Patients and Citizens A Framework for Action*. Retrieved from https://assets.publishing.service.gov.uk/government/uploads/system/uploads/attachment_data/file/384650/NIB_Report.pdf.

6 NHS England. (2019). The NHS Long Term Plan Long-Term. Retrieved from https://www.longtermplan.nhs.uk/wp-content/uploads/2019/01/nhs-long-term-plan.pdf. https://www.longtermplan.nhs.uk/.

7 This logic is very cybernetic, it relies on an assumption that digital health tools view humans as stable, controllable entities capable of maintaining an equilibrium by responding to changes in health outputs by altering inputs accordingly (Cheney-Lippold 2017).

8 This is an example of Butler’s ideas of power that combine those of Foucault with those of psychoanalysis to argue that subjects only come into being through power. Thus, without the power of the data of digital health tools, the digital self would never have come into being and is thus unaware of how it has been formed by power (Nagington et al. 2016).

9 Similar arguments have been made about the US healthcare system, where the transition has been from patient to consumer (Goldstein and Bowers 2015).

10 For Marxists this denial would be a form of false consciousness where an increase in empowerment does not necessarily translate into an actual increase in power in terms of the ability to influence outcomes (Riger 1993).

11 We are grateful to Effy Vayena for bringing this connection to our attention.

12 Further information about the NHS App can be viewed here: https://www.nhs.uk/using-the-nhs/nhs-services/the-nhs-app/. [Accessed May 2019]

13 In other words, the empowerment narrative assumes that what matters is a Kantian deontological definition of autonomy which solely focuses on will, but the reality is that empowerment in healthcare relies on individuals being autonomous in both the Kantian sense and in the utilitarian action-focused sense advocated by Mills (Komrad 1983).

14 For example, a study by Emil Chiauzzi and colleagues (2016) on factors influencing feelings of empowerment amongst patients using *Patients Like Me* surveyed 3988 participants and found that those who were older, male, more educated and privately insured all reported significantly higher levels of empowerment.

15 This type of constant shifting is referred to as modulation. Both Michel Foucault and Gilles Deleuze talk about how power and surveillance influence modulation but it is Deleuze’s theory that applies here as he argued that in control societies, modulations happen in opaque networks that are invisible to individual citizens by making surveillance forces less overt and physical, and more abstract and numerical (Galic et al. 2017). Thus, individuals become subject to a regime of numbers where the health of their digital self is constantly shifting depending on hours slept, steps taken, calories consumed etc. (Owens and Cribb 2017). Furthermore, as the devices that generate these data that control the constant modulation of the digital self, are normally held on the individual’s person, this can be seen as surveillance that colonises the user’s lifeworld as defined by Jürgen Habermas (Owens and Cribb 2017).

16 This is because as our health care system becomes increasingly *dependent* on these complex interacting multi-agent systems, the tools available to us to shape the available options and courses of actions for entire segments of the population increases, and thus the temptation to do so must be resisted even more forcefully (Floridi 2016b).

17 Poole, N. (2016). A common law right to autonomy of treatment. Retrieved from https://www.rcseng.ac.uk/news-and-events/blog/the-right-to-autonomy-of-treatment-is-a-common-law-right/.

18 For now, it is assumed that such a tool has, following rigorous testing, been proven to be clinically safe and effective. A discussion of how the governance of the mHealth ecosystem needs to be designed to ensure that this is *always* the case is beyond the scope of this article, but we recognise that it is a live and important issue.

19 Allowing for some kind of manipulation of the choice architecture in this manner is key to avoiding the ‘dilemma’ of tolerance where it is possible that a zero form of pragmatic paternalism can become hyper-protective and thus counterproductive to wellbeing. Positioning mHealth tools as digital companions makes possible a fruitful resolution between paternalism and toleration in view of a balanced respect for an agent’s choices by forcing the agent (person, group or artificial agent) to exercise their autonomy consciously (Floridi 2016b).

20 To be more formal, the person, group or artificial agent using the mHealth tool is variable (A) and the person, group or artificial agent providing the advice is variable (B) and their interaction is being mediated by variable (Z) ‘mHealth tool’. Thus, the logic applies to any combination of person, group or artificial agent interacting with person, group or artificial agent, via an mHealth tool (Floridi 2016a).

21 All information sourced from the website of the Cystic Fibrosis Trust: https://www.cysticfibrosis.org.uk/life-with-cystic-fibrosis. [Accessed February 2019]

22 See http://www.cfbuzz.org/app/. [Accessed May 2019]

23 This distinction is important because, in the ethics of information too, information can be both qualitative and quantitative (Floridi 2010). In this context, the even distribution of information is referring to the *qualitative* aspect of information (relevance) to which both parties, facilitated by the digital companion third variable, have equal access to deal with the decision in hand. This does not mean that both parties have the *same amount* (quantitative) information. The HCP will still know *more* about the theory of the medical intervention being suggested, and the individual will still know *more* about what it is like to live with their symptoms, creating an equal distribution in this quantitative sense of information is unnecessary in this context.

24 The authors wish to thank the journal’s anonymous reviewers for their careful and insightful comments that led to a much better final version.
